# A risk signature of four aging-related genes has clinical prognostic value and is associated with a tumor immune microenvironment in glioma

**DOI:** 10.18632/aging.203146

**Published:** 2021-06-10

**Authors:** Haitao Luo, Chuming Tao, Xiaoyan Long, Kai Huang, Xingen Zhu

**Affiliations:** 1 Department of Neurosurgery, The Second Affiliated Hospital of Nanchang University, Nanchang, Jiangxi Province, China; 2 East China Institute of Digital Medical Engineering, Shangrao, Jiangxi Province, China; 3 Institute of Neuroscience, Nanchang University, Nanchang, Jiangxi Province, China

**Keywords:** aging-related genes, prognostic biomarker, tumor immune microenvironment, glioma

## Abstract

An accumulation of studies has indicated aging to be a significant hazard factor for the development of tumors. Cellular senescence is positively associated with aging progress and aging-related genes (AGs) can regulate cellular senescence and tumor malignancy. While the association between AGs and the prognosis of patients with glioma is still unclear. In our study, we initially selected four survival-associated AGs and performed consensus clustering for these AGs based on The Cancer Genome Atlas (TCGA) database. We then explored the potential biological effects of four selected AGs. A prognostic risk model was constructed according to four selected AGs (LEP, TERT, PON1, and SSTR3) in the TCGA dataset and Chinese Glioma Genome Atlas (CGGA) database. Then we indicated the risk score was an independent prognostic index, and was also positively correlated with immune scores, estimate score, immune cell infiltration level, programmed death ligand 1 (PD-L1) expression, and expression of proinflammatory factors in patients with glioma. Finally, we performed the RT-qPCR and immunohistochemistry assay to validate our bioinformatics results. Thus, this study indicated the risk model was concluded to possibly have potential function as an immune checkpoint inhibitor and to provide promising targets for developing individualized immunotherapies for patients with glioma.

## INTRODUCTION

Glioma is an aggressive brain tumor with high recurrence rates [1–3]. Universal treatment strategies for glioma involves surgical resection with postoperative chemotherapy and radiotherapy, but the clinical prognoses for patients suffering from glioma still remain poor due to its lethal malignancy [4–7]. Nowadays, a need still exists to find more advanced treatments for glioma.

Aging is an essentially universal characteristic of living organisms, is considered to involve a progressive decline of internal cellular functions and is a hot spot in tumor research [8–10]. Tumor, like the other diseases of aging, become much more prevalent beginning at around the midpoint of life. Cellular senescence plays significant role in contributing the aging progress and developing of tumor, while the mechanisms of it on tumor are extremely complex, which can both stimulate and suppress tumor malignancy [11–13]. For example, some *in vivo* experiments indicated cellular senescence could restrict tumorigenesis in early-stage prostate cancer and Braig et al. revealed H3K9me-mediated cellular senescence as a novel mechanism to suppress the formation of lymphomas [14, 15]. While some studies indicated an obvious ability of injected senescent fibroblasts to stimulate the proliferation of human epithelial tumor cells in immunocompromised mice [16, 17], which was closely associated with the senescence-associated secretory phenotype (SASP) [18]. Aging-related genes (AGs) can regulate cellular senescence and play a key role in tumor malignancy [11, 19]. There is, however, limited knowledge about the relationships between AGs and the prognosis of patients with glioma.

In our study, we selected four survival-associated AGs and performed consensus clustering for these AGs based on The Cancer Genome Atlas (TCGA) database. We then explored the potential biological effects of four selected AGs. Then a prognostic risk model was constructed, we indicated the risk score was an independent prognostic index, and was positively correlated with immune scores, estimate score, immune cell infiltration level, programmed death ligand 1 (PD-L1) expression, and proinflammatory factors expression in patients with glioma. Finally, we performed some laboratory experiment to validate our bioinformatics results. Our research revealed their underlying implication as biomarkers for predicting clinical prognosis of patients with glioma.

## RESULTS

### Selection the AGs association with the prognostic of patients with glioma

**Figure 1:**
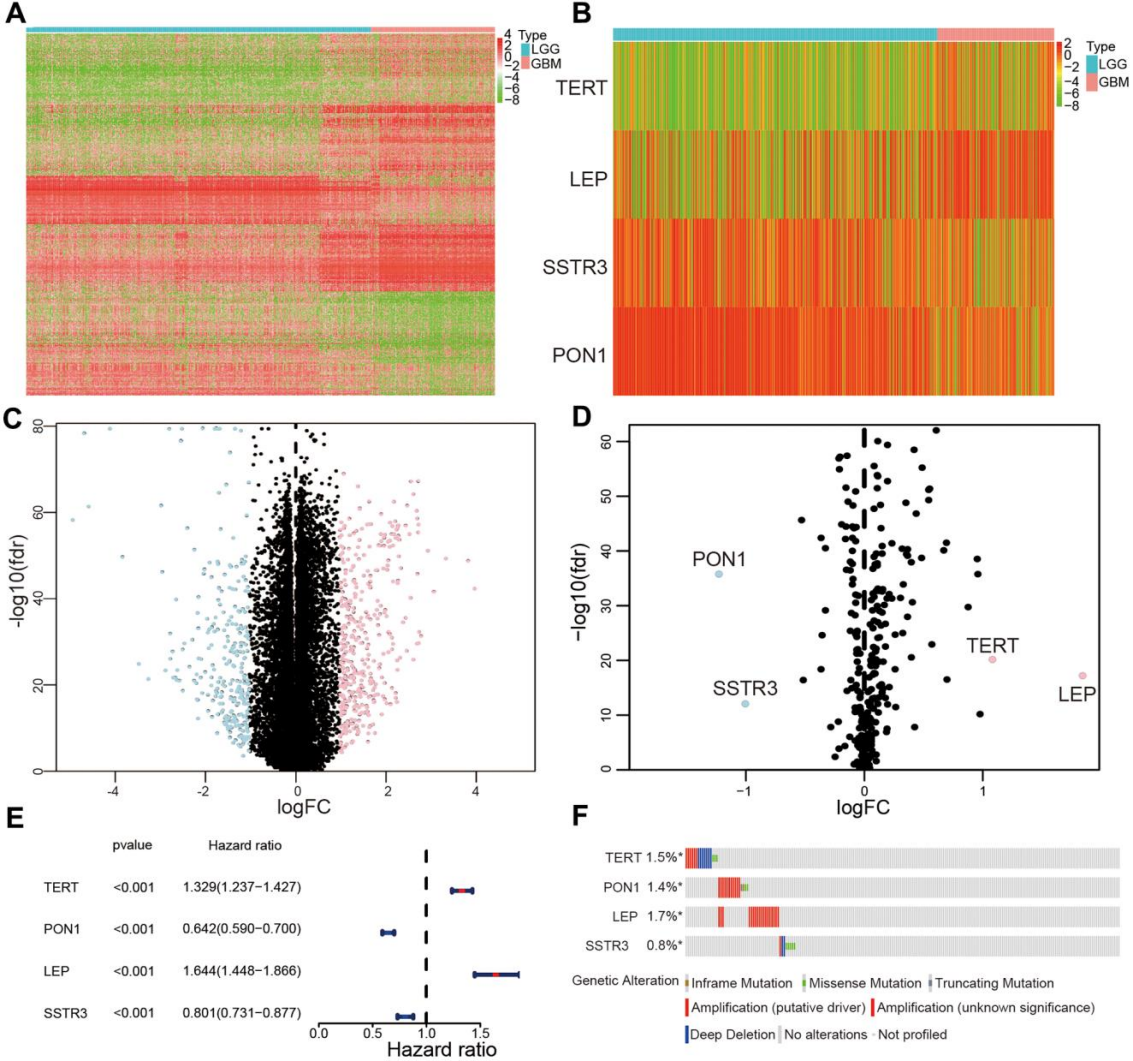
**Selection of differentially expressed and survival-associated AGs based on TCGA datasets.** (**A**) Heatmap of all genes with significant differences between lower-grade glioma (LGG) and glioblastoma (GBM) samples. (**C**) Volcano plot of all genes based on TCGA datasets, light blue represented downregulated of genes, and pink represented upregulated of genes. (**B**) Differentially expressed AGs are showed in heatmap between LGG and GBM samples. (**D**) Volcano plot of the four selected differentially expressed AGs, light blue represented downregulated of AGs, and pink represented upregulated of AGs. (**E**) Forest plot of the four differentially expressed AGs. (**F**) Genetic changes of the four survival-associated AGs.

To find differentially expressed AGs, we initially selected 676 differentially expressed genes based on TCGA database (Figure 1A, 1C). Then we identified 4 differentially expressed AGs from 676 differentially expressed genes: LEP and TERT were upregulated while PON1 and SSTR3 were downregulated (Figure 1B, 1D). We then found that these AGs were correlated with the prognosis of patients with glioma (*P* < 0.01, Figure 1E). We also identified that the frequency of these survival-associated AG genetic alterations (< 1.8%, Figure 1F).

### Consensus clustering for four survival-associated AGs and with the prognoses of patients with glioma

**Figure 2:**
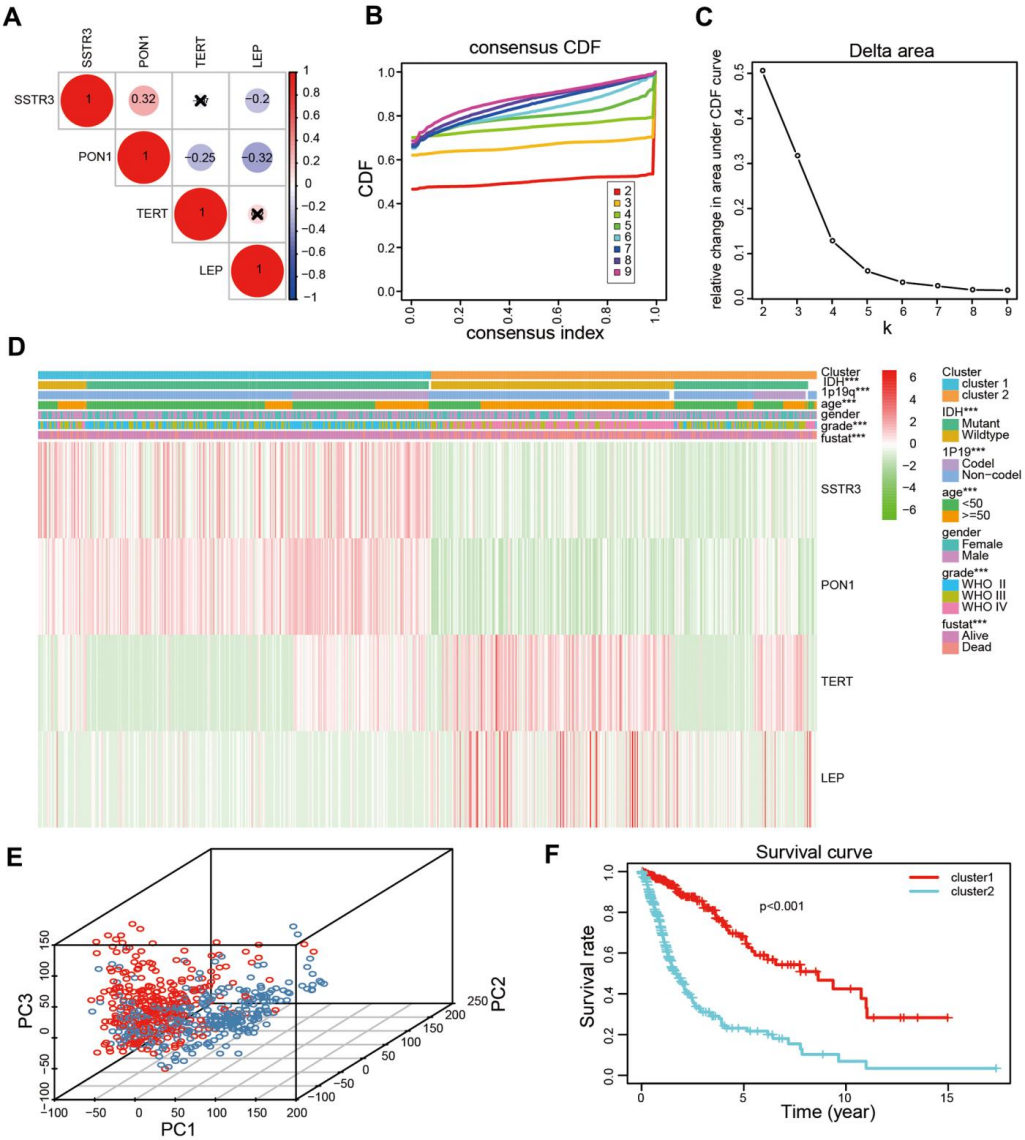
**Two categories of patients based on distinct clinical characteristics and the OS according to the expression levels of the four survival-associated AGs in the TCGA datasets.** (**A**) Spearman’s correlation analysis of the four survival-associated AGs (LEP, TERT, PON1, and SSTR3) in the TCGA datasets. (**B**) The relative change of the area under the cumulative distribution function (CDF) for K =2-9. (**C**) The consistency clustering CDF curve for K = 2-9. (**D**) Clinical characteristics of two clusters identified based on the expression levels of the four survival-associated AGs in the TCGA datasets. (**E**) Principal component analysis (PCA) for the total mRNA expression profile based on TCGA datasets. (**F**) Kaplan-Meier (KM) curves for 628 cluster1 and cluster2 glioma patients based on the TCGA datasets. * *P* < 0.05, ** *P* < 0.01, and *** *P* < 0.001.

To explore the association of four survival-associated AGs, we performed correlation analysis according to their mRNA expression level in the TCGA datasets. Our results revealed the expression of PON1 was crucially positive associated with SSTR3 in glioma, while there were a crucial negative association between the expression of PON1 and TERT, and the expression of LEP was negatively associated with PON1 and SSTR3 (Figure 2A). Consensus clustering analysis was used to sort samples into subtypes based on the expression profiles of the above-identified four survival-associated AGs in the TCGA datasets. The resulting cumulative distribution function (CDF) curves and SigClust analysis indicated a K value of = 2 (Figure 2B, 2C and Supplementary Figure 1), categorization of two subtypes (cluster1 (n = 317) and cluster2 (n = 311)) on the basis of different expression levels of the four survival associated AGs. The expression levels of the four selected AGs were statistically different between the two subtypes, which showed cluster2 with the upregulated expression levels of the risk factors (TERT and LEP) and clsuter1 with the upregulated expression of protective factors (SSTR3 and PON1, Figure 2D). Then we further revealed the gene expression profiles between the two subtypes were differentiated well by using principal component analysis (PCA, Figure 2E). The Kaplan-Meier (KM) curves indicated a poor prognosis for the samples in cluster2 (*P* < 0.001, Figure 2F). Furthermore, high grade, old age, mutant-type isocitrate dehydrogenase (IDH) status,1p19q non-codeletion status were presented in cluster2 than cluster1 (Figure 2D and Supplementary Table 1).

### Consensus clustering analysis revealed the potential cellular biological effects of four survival-associated AGs

**Figure 3:**
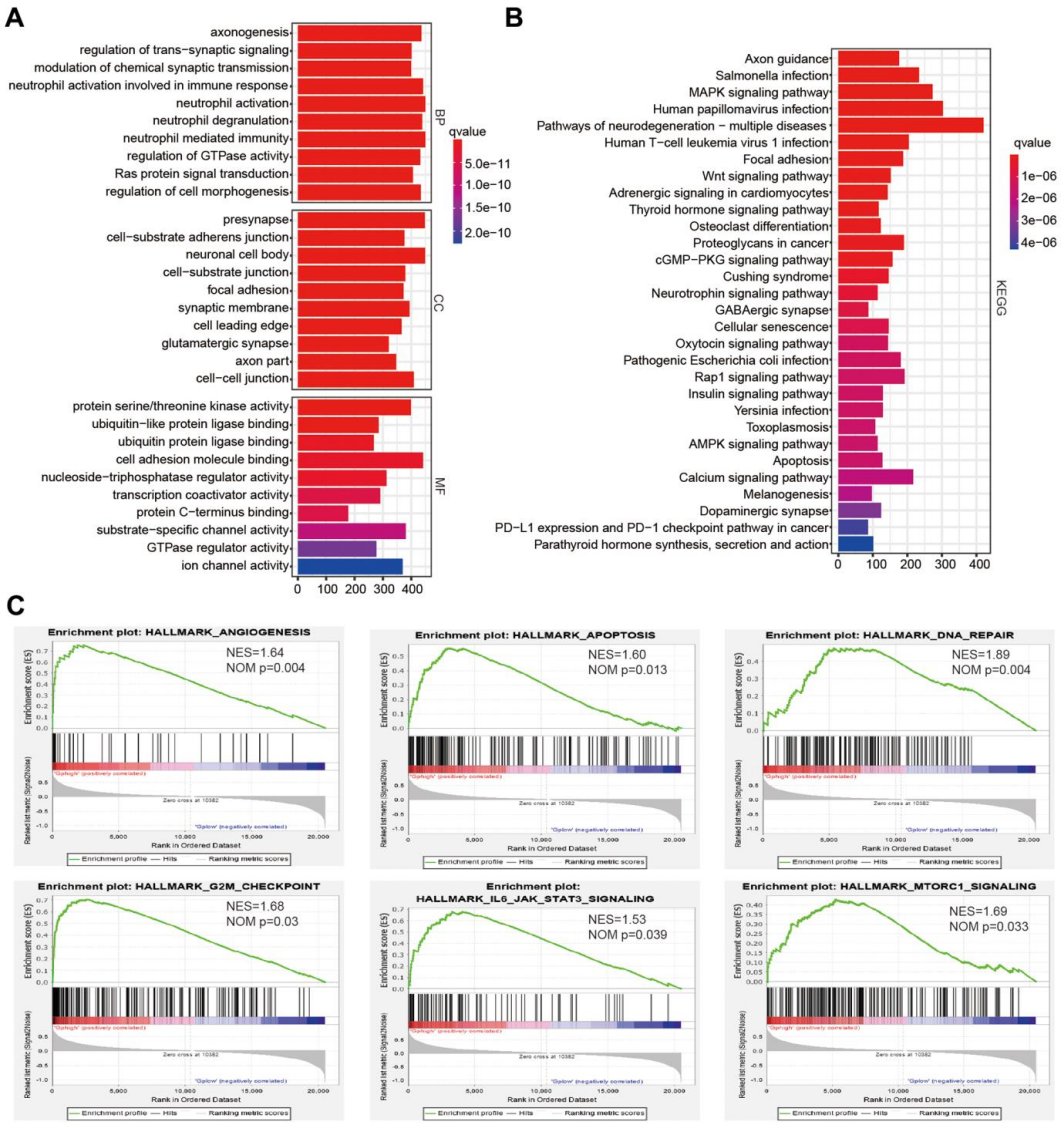
**The potential biological functions of four selected associated-survival genes.** (**A**, **B**) Functional annotations of differentially expressed genes in clsuter2 compared with cluster1 based on TCGA datasets determined from Geno Ontology (GO) and Kyoto Encyclopedia of Gene and Genomes (KEGG) pathway analyses. (**C**) Malignancy hallmarks positively enriched in cluster2 determined using gene set enrichment analysis (GSEA) in the TCGA datasets.

Since cluster2 presented the low OS, the malignancy-related mechanisms were further explored in this subtype. We selected differentially expressed genes between cluster2 and cluster1, and analyzed some biological processes significantly correlated with cluster2. Compared with cluster1, genes whose products are involved in neutrophil degranulation, neutrophil activation, ras protein signal transduction and regulation of cell morphogenesis were positively enriched in cluster2 (Figure 3A). A Kyoto Encyclopedia of Genes and Genomes (KEGG) analysis also revealed a crucial positive enrichment of genes involved in human papillomavirus infection, the wnt signaling pathway, cellular senescence, and the AMPK signaling pathway (Figure 3B). In addition, we further showed malignant hallmarks by carrying out a gene set enrichment analysis (GSEA), which indicated that the IL6 JAK STATS signaling (NES = 1.53, normalized *P* = 0.039), apoptosis (NES = 1.60, normalized *P* = 0.013), DNA repair (NES = 1.89, normalized *P* = 0.004), and G2M checkpoint (NES = 1.68, normalized *P* = 0.03) were significantly positively associated with cluster2 (Figure 3C). In conclusion, our results might provide novel insights for cellular biological function related to four survival-associated AGs.

### Identification of the prognostic value of selected AGs and risk model derived from four select AGs

**Figure 4:**
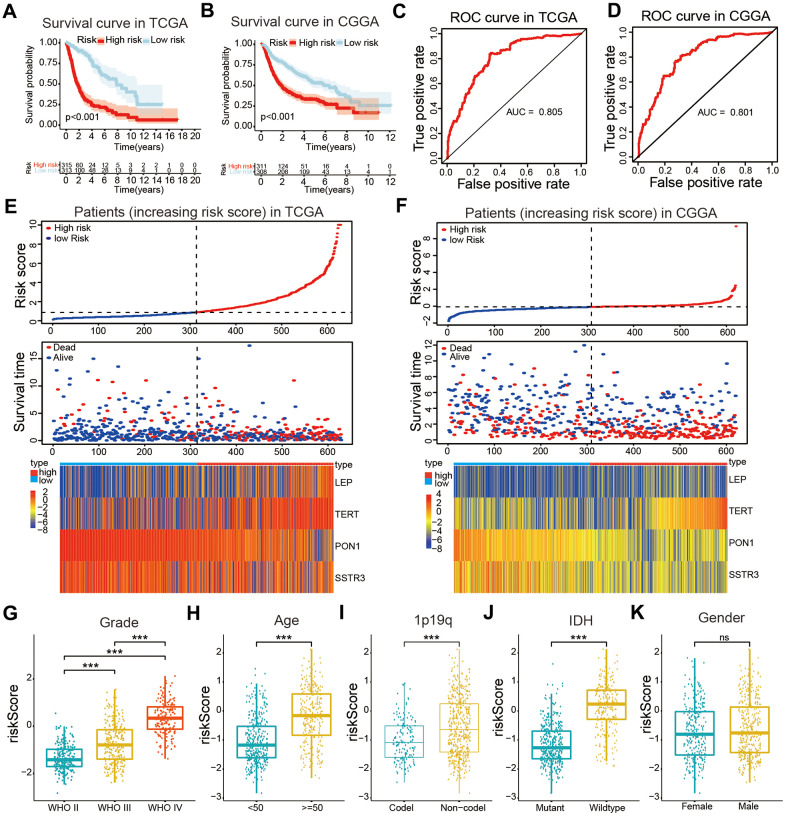
**Construction of a risk model and the association of the risk model with clinical characteristics of patients with glioma.** (**A**, **B**) Kaplan-Meier (KM) curves for overall survival (OS) prediction based on the training (TCGA) and validation (CGGA) datasets. (**C**, **D**) Receiver operating characteristic (ROC) curves for the risk model both in the training and validation datasets. (**E**, **F**) Risk plot distribution, survival status of patients, and heatmap of expression of included genes in the training and validation datasets. (**G**–**K**) Relationships between the risk score and clinical characteristics (grade, age, 1p19q status, IDH status, and gender) of patients with glioma. Non-significant (ns) *P* > 0.05, * *P* < 0.05, ** *P* < 0.01, and *** *P* < 0.001.

The least absolute shrinkage and selection operator (Lasso) Cox regression algorithm was used to build a prognostic risk model according to the expression levels of the four selected AGs in the TCGA datasets, and coefficients were obtained to calculate the risk scores for each patient with glioma (Supplementary Figure 2). We sorted glioma samples into two subtypes by the median risk scores. The KM curves revealed that the samples in the high-risk categories had a poorer OS than did those in the low-risk categories based on training and validation databases (*P* < 0.001, Figure 4A, 4B). The area under the time-dependent receiver operating characteristic (ROC) curve (AUC) values were calculated to assess the value of our four-AG risk model which was 0.805 for the TCGA dataset, 0.801 for the Chinese Glioma Genome Atlas (CGGA) datasets (Figure 4C, 4D). These results showed the accuracy of our four-AGs risk model for glioma prognosis. The risk plot distribution, survival status of patients with glioma, and heatmap of the expression of included genes were determined based on the TCGA and CGGA databases (Figure 4E, 4F).

### Positive correlations of the risk model with the OS and clinical characteristics of patients with glioma

**Figure 5:**
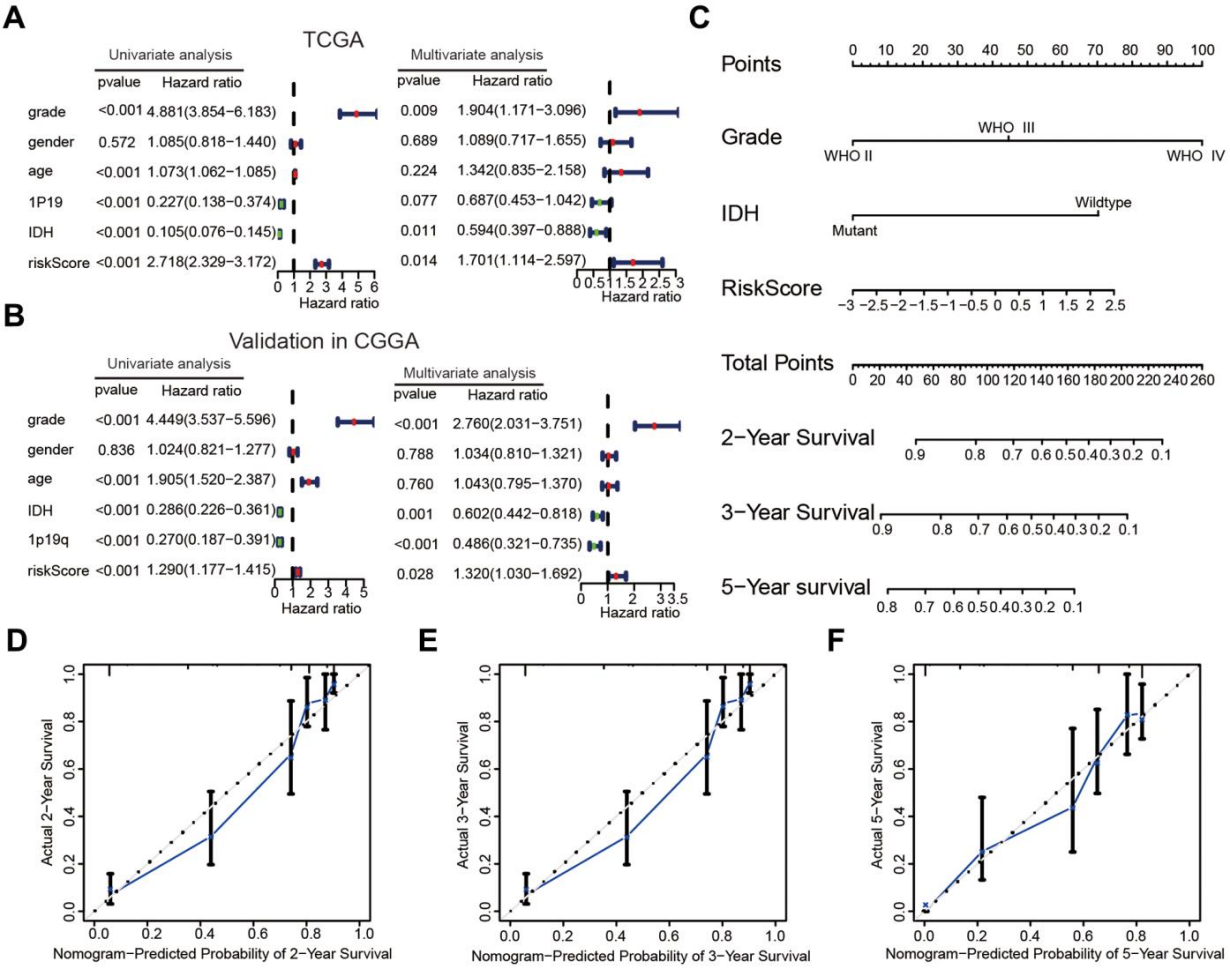
**The risk score could serve as an independent prognostic index and predict the prognoses of patients with glioma.** (**A**, **B**) Univariate and multivariate Cox analysis of clinical characteristics and molecular features based on the training (TCGA) and validation (CGGA) datasets. (**C**) Nomogram based on the WHO grade, IDH status and risk score for providing prognoses of patients with glioma in the TCGA datasets. (**D**–**F**) Calibration plots used to indicate that the nomogram could effectively predicted the 2-,3-, and 5- years prognoses of patients with glioma.

The correlations between the risk scores and clinical characteristics of patients with glioma were examined using the Wilcoxon test, which showed significant differences between risk scores in groups stratified by the Word Health Organization (WHO) tumor grade (*P* < 0.001), age (*P* < 0.001), 1p/19q status (*P* < 0.001) and IDH status (*P* < 0.001, Figure 4G–4K). Univariate Cox regression analyses revealed that the WHO grade, age, IDH status, 1p/19q status, and risk score were significantly correlated with prognosis of patients in the TCGA datasets. Multivariate Cox regression analyses showed that the WHO grade (*P* < 0.01), IDH status (*P* < 0.05), and risk score (*P* < 0.05) remained crucially associated with the OS of patients (Figure 5A). Similar conclusions were made based on the results of the multivariate Cox regression analysis using the validation dataset (Figure 5B), which revealed positive associations of the WHO grade (*P* < 0.001), IDH status (*P* < 0.01), 1p19q status (*P* < 0.001), and risk scores (*P* < 0.05) with prognosis. Therefore, the risk scores derived from the four selected AGs indicated a good prognostic performance for this set of AGs and that they could represent an independent prognostic index for glioma. A nomogram was then constructed for providing a prognosis of glioma that integrated tumor grade, IDH status, and risk scores based on the TCGA database (Figure 5C), and the C-index for survival prediction was 0.828. The calibration plots for providing prognoses of patients at 2-, 3-, and 5- years revealed an optimal agreement between the nomogram prediction and the actual observed outcomes (Figure 5D–5F). The results showed this four-AGs risk model to have accurate predictive value for prognosis and clinicopathological features.

### The association between the risk model and the immune cell infiltration level in glioma

**Figure 6:**
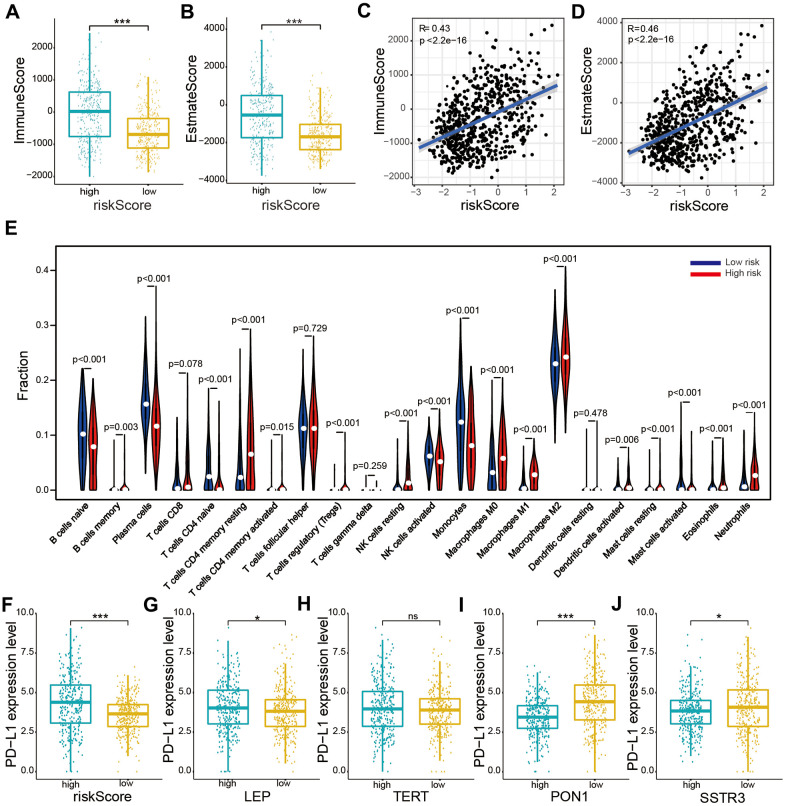
**Relationships between the risk score and immune cell infiltration levels, and PD-L1 expression levels based on TCGA datasets.** (**A**, **B**) Comparison of immune and estimate scores according to two subtypes grouped by the median risk score. (**C**, **D**) Relationship between the risk score and immune scores and the estimate score. (**E**) The violin plot showed distribution of the ratio differentiation of 22 kinds of immune cells in glioma samples according to two subtypes grouped by the median risk score. (**F**–**J**) Distribution of PD-L1 expression levels based on the risk score (**F**) and expression levels of the four selected AGs (LEP (**G**), TERT (**H**), PON1 (**I**), and SSTR3 (**J**)) of patients with glioma in the TCGA datasets. Non-significant (ns) *P* > 0.05, * *P* < 0.05, ** *P* < 0.01, and *** *P* < 0.001.

To explore the occurrence of any association between the risk score, immune score, and estimate score, the estimate formula was performed to calculate the immune score and estimate score of patients with glioma based on the TCGA dataset. The high-risk subtype was associated with a higher immune score and estimate score than was the low-risk subtype (*P* < 0.001, Figure 6A, 6B). Furthermore, our results also showed crucially positive associations of risk score with immune score (*R* = 0.43, *P* < 0.001, Figure 6C) and estimate score (*R* = 0.46, *P* < 0.001, Figure 6D) in glioma samples.

We evaluated the composition of 22 important immune fractions of samples based on the TCGA dataset using the CIBERSORT formula to explore the compositional fraction of 22 types of immune cells between high-risk and low-risk subtypes (Figure 6E). The results revealed that patients with glioma in the high-risk subtype included a higher fraction of memory B cells (*P* < 0.05), CD4 memory resting T cells (*P* < 0.001), CD4 memory activated T cells (*P* < 0.001), regulatory T (Tregs) cells (*P* < 0.001), resting NK cells (*P* < 0.001), M0 macrophages (*P* < 0.001), M1 macrophages (*P* < 0.001), M2 macrophages (*P* < 0.001), activated dendritic cells (*P* < 0.01), resting mast cells (*P* < 0.001), activated mast cells (*P* < 0.001), eosinophils (*P* < 0.01), and neutrophils (*P* < 0.001) than did those in the low-risk subtype. While naïve B cells, plasma cells, naïve CD4 T cells, activated NK cells, monocytes, and activated mast cells showed the opposite result (*P* < 0.001). Our results showed that the risk score was statistically positively correlated with immune cell infiltration levels in glioma.

### Association of PD-L1 expression with the risk score and the four selected AGs

To explore the relationship between PD-L1 and the four selected AGs, expression levels of PD-L1 were calculated for the high-risk and low-risk subtypes based on the TCGA database. The PD-L1 expression was found to be upregulated in the high-risk subtypes compared to that in the low-risk subtypes (Figure 6F). Furthermore, the PD-L1 expression levels showed a positive correlation with risk factors (LEP and TERT, Figure 6G, 6H), while a crucially negative relationship was noted with protective factors (PON1 and SSTRS, Figure 6I, 6J).

### Association of the six types of immune cells with the four selected AGs in the risk model

**Figure 7:**
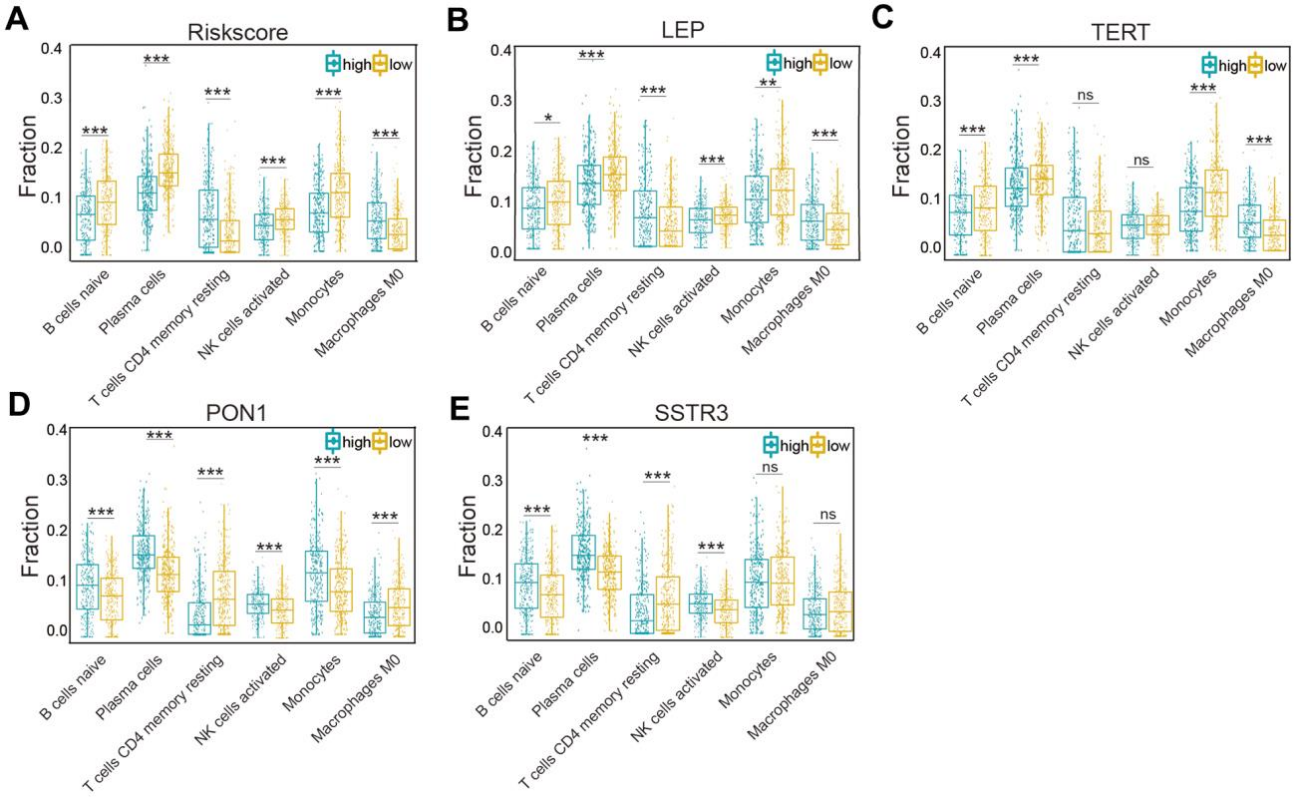
**Association of six types of immune cells with the risk score and four selected AGs in the TCGA datasets.** (**A**) Distributions of the six types of immune cells (naïve B cells, plasma cells, CD4 memory resting T cells, NK activated cells, monocytes, and M0 macrophages) in the two subtypes grouped by the median risk score. (**B**–**E**) Comparison of the six types of immune cells (naïve B cells, plasma cells, CD4 memory resting T cells, NK activated cells, monocytes, and M0 macrophages) according to LEP (**B**), TERT (**C**), PON1 (**D**), and SSTR3 (**E**) expression levels. Non-significant (ns) *P* > 0.05, * *P* < 0.05, ** *P* < 0.01, and *** *P* < 0.001.

According to the associations of the risk score and the six types of immune cells (Figure 7A), the relationships between these immune cells and the four selected AGs were investigated. Glioma patients of the subtype expressing high levels of LEP had a lower fraction of naïve B cells, plasma cells, NK activated cells, and monocytes cells than did those of subtype expressing low levels of LEP, while the opposite was the case for CD4 memory resting T cells and M0 macrophages (*P* < 0.05, Figure 7B), in accord with the results grouped by the risk score. Similarly, the results for TERT, except for the CD4 memory resting T cells and NK activated cells (*P* > 0.05), were also in accord with the risk score results (*P* < 0.01, Figure 7C). But the results for PON1 and SSTR3, as protective factors, were not in accord with the risk score results; glioma patients of the high-LEP-expression subtype included a higher fraction of naïve B cells, plasma cells, NK activated cells, and monocytes than did those of low-LEP-expression subtype, while CD4 memory resting T cells and M0 macrophages showed the opposite result (*P* < 0.05, Figure 7D). Similarly, the results for SSTR3, except for M0 macrophage and monocytes (*P* > 0.05), were in contrast with the risk score results (*P* < 0.01, Figure 7E). The four selected AGs (LEP, TERT, PON1, and SSTR3) were concluded to have important functions in immune cell infiltration of glioma.

### Association of proinflammatory factors with the risk score and the four selected AGs

An accumulation of research has indicated chronic inflammation associate with cellular senescence to have an important function in immune cell infiltration and major proinflammatory factors, including interleukin-1α (IL-1α), interleukin-1β (IL-1β), interleukin-6 (IL-6), interleukin-8 (IL-8) and interleukin-18 (IL-18) [20, 21]. In our study, we explored the association of major proinflammatory factors with the risk score and the four selected AGs, and showed the expressing features of eight major proinflammatory factors based on the TCGA datasets (Supplementary Figure 3). Our results revealed that the levels of expression of IL-1α, IL-1β, IL-6, IL-8, and IL-18 in the high-risk groups were statistically higher than those in the low-risk groups (*P* < 0.001, Table 1), in accord with the risk score results. This study also revealed that IL-1α, IL-1β, IL-6, IL-8, and IL-18 were expressed at higher levels in the high-LEP-expression group than in the low-LEP-expression group (*P* < 0.05, Table 1). And the results for TERT showed that, except for IL-1α (t = 1.640, *P* = 0.104), IL-1β (t = -2.646, *P* < 0.001), and IL-18 (t = -2.827, *P* < 0.001), the other interleukins were expressed at high levels in the high-TERT-expression group (*P* < 0.05, Table 1). The PON1 and SSTR3, as protective factors, the results showed that IL-1α, IL-1β, IL-6, IL-8, and IL-18 were expressed at lower levels in the high expression group than in the low expression group (*P* < 0.05, Table 1).

**Table 1:** Correlation of proinflammatory factors with the risk score and gene of the risk model in the TCGA dataset.

**Genes**	**TERT**		**PON1**		**LEP**		**SSTR3**		**Risk score**	
	**t**	***P***	**t**	***P***	**t**	***P***	**t**	***P***	**t**	***P***
**IL-1α**	1.640	0.104	-6.382	<0.001	3.187	<0.01	-3.060	<0.01	3.747	<0.001
**IL-1β**	-2.646	<0.001	-4.481	<0.001	2.367	<0.05	-1.276	0.203	4.231	<0.001
**IL-6**	2.291	0.02	-9.763	<0.001	5.730	<0.001	-4.167	<0.001	8.591	<0.001
**IL-8**	5.958	<0.001	-9.244	<0.001	5.637	<0.001	-4.045	<0.001	9.270	<0.001
**IL-18**	-2.827	<0.01	-7.259	<0.001	2.96	<0.01	-7.367	<0.001	5.704	<0.001

t: t value of student’s test; *P*: *P*-value of student’s t test.

### The mRNA and protein expression levels of four selected AGs in glioma

**Figure 8:**
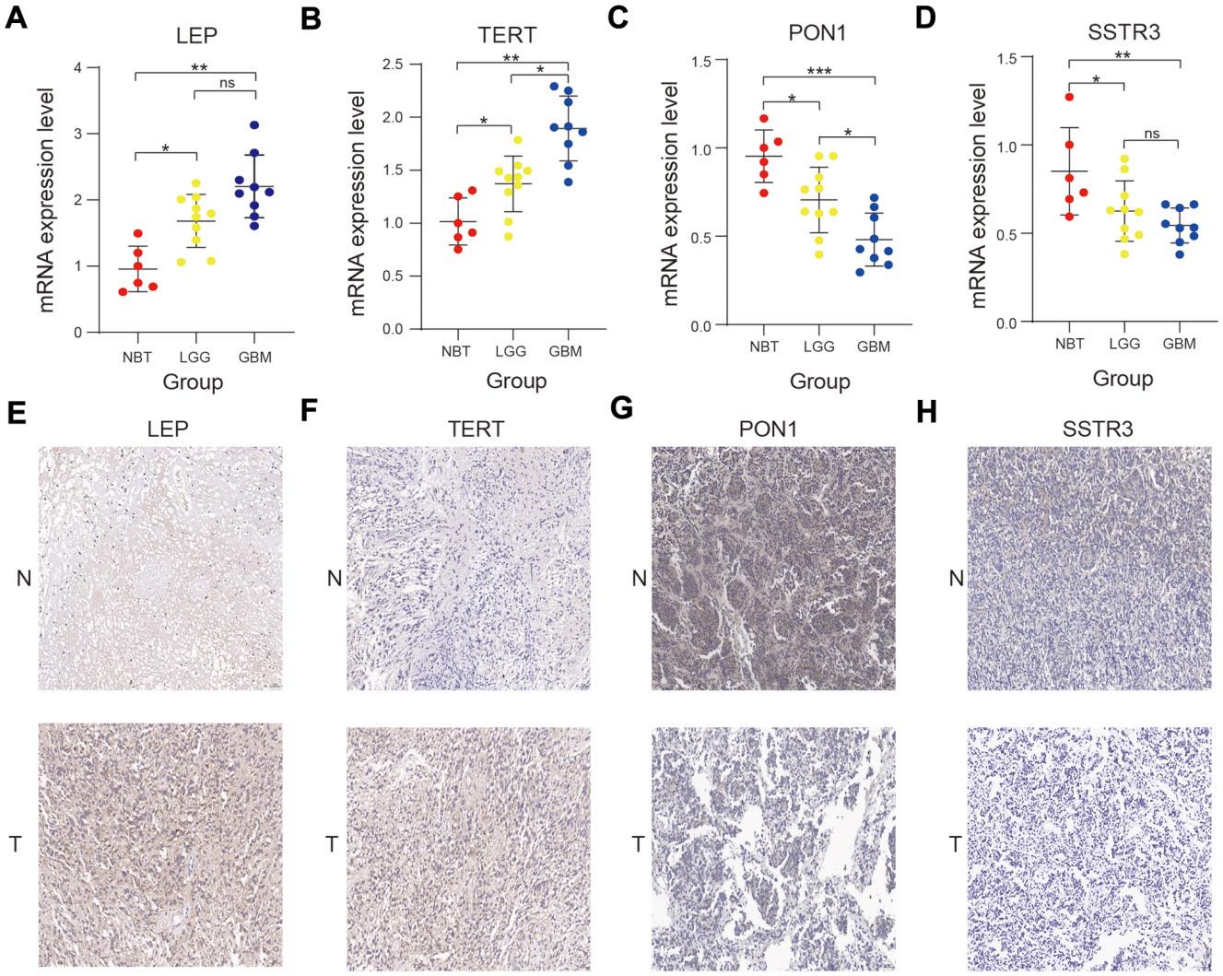
**Validation of the bioinformatics results using RT-qPCR and immunohistochemistry assay.** (**A**–**D**) Comparison of LEP (**A**), TERT (**B**), PON1 (**C**), and SSTR3 (**D**) mRNA expression levels in normal brain tissue (NBT), lower-grade glioma (LGG), and glioblastoma (GBM) tissues by RT-qPCR assay. (**E**–**H**) Comparison of the protein expression of LEP (**E**), TERT (**F**), PON1(**G**) and SSTR3 (**H**) in NBT and glioma tissue by immunohistochemistry assay. Non-significant (ns) *P* > 0.05, * *P* < 0.05, ** *P* < 0.01, and *** *P* < 0.001.

We performed the Real-time quantitative polymerase chain reaction (RT-qPCR) and immunohistochemistry assay to validate the bioinformatics results. The RT- qPCR assay showed that the four AGs (LEP, TERT, PON1, and SSTR3) were expressed to different extents in normal brain tissue (NBT), lower-grade glioma (LGG) and Glioblastoma (GBM) tissue in mRNA expression level (Figure 8A–8D), and the immunohistochemistry assay revealed that the protein expression of four AGs were also expressed to different extents in NBT and glioma tissue (Figure 8E–8H), in accord with the bioinformatics results.

## DISCUSSION

There are currently many pieces of evidences of age-dependent changes being correlated with proinflammatory nature and involved in chronic inflammatory microenvironment [22–24]. Increasing in inflammation and immune cell infiltration levels with age-dependent changes may contribute to formation and malignancy of tumors [25], while the functions of them in the malignancy of glioma are still unclear. Exploring the molecular mechanisms of AGs is important for identifying the role of age-dependent changes in glioma. Few studies have to date systematically investigated the molecular mechanisms of AGs in glioma and the association between the expression profile of AGs and the OS of patients with glioma.

In our study, we performed a systematic analysis to select the differentially expressed and survival-associated AGs. Consensus clustering analysis was used to sort glioma samples into two clusters according to the expression profile of four survival-associated AGs (TERT, LEP, PON1, and SSTR3). The cluster1/2 subtypes could affect prognosis and clinical characteristics of patients with glioma, and revealed strong correlations with some significant biological processes and signaling pathways, including human papillomavirus infection, the wnt signaling pathway, cellular senescence and the AMPK signaling pathway. We then established a risk model with the four selected AGs and showed that the risk score could be regarded as an independent prognostic index for predicting the prognosis of patients with glioma based on both training (TCGA) and validation (CGGA) datasets. In our risk model, TERT and LEP acted as risk factors, while PON1 and SSTR3 acted as protective factors. For TERT, it had been shown the promoter mutations were correlated with poor prognosis and shorter survival of patients with glioma [26, 27], and some researches also identified that TERT promoter mutations was an independent prognostic factor in the other human cancer [28, 29]. Yeh et al. found LEP expressed at higher levels in glioma than in astrocytes, and indicated that high levels of expression of LEP could promote glioma cell migration and invasion by increasing the MMP-13 expression levels [30]. Zhang et al. identified low LEP expression level was correlated with short OS and low complete remission rate in acute myeloid leukemia [31]. The molecular mechanisms of PON1 and SSTR3 in glioma remained ambiguous. Li et al. found PON1 inhibited the proliferation and migration of renal cancer cells and Ding et al. reported PON1 could be used as a biomarker for evaluating the invasion of hepatocellular carcinoma [32, 33]. Hu et al. indicated low expression in gastric cancer and expressed SSTR3 could inhibit gastric cancer cell proliferation and induce cell apoptosis [34].

Interestingly, our Gene Ontology (GO) analysis results showed that neutrophil activation involved in immune response, neutrophil activation and neutrophil mediated immunity were enriched in cluster2. Neutrophils have been shown to have important functions for the innate immune response and to be involved in immune cell functions [35–37]. Therefore, we explored the association of risk score with immune score, PD-L1 expression, and compositional fraction of 22 types of immune cells in patients with glioma based on TCGA datasets. The PD-L1 expression levels and immune score of the high-risk subtypes were crucially higher than those of the low-risk subtypes, and positive associations between the risk score and the levels of most of the immune cells were observed: high-risk subtypes contained higher fractions of CD8 T cells, CD4 memory resting T cells, CD4 memory activated T cells, regulatory T cells, resting NK cells, M0 macrophages, M1 macrophages, M2 macrophages, activated dendritic cells, resting mast cells, eosinophils and neutrophils than did the low-risk subtypes. Our study also showed a significant positive association of the risk score with immune cell infiltration, and its correlation with the growth and differentiation of macrophages and T cells. Some SASP components play a crucial role in the development of inflammation, which is relevant to the potential functions of senescent cells in tumor malignancy [25, 38], and the proinflammatory factors of the SASP can influence the immune cell infiltration levels [39, 40]. So we also substantiated a crucially positive correlation of the risk score with IL-1α, IL-1β, IL-6, IL-8, and IL-18 expression levels in patients with glioma. We substantiated the idea of TERT and LEP being the risk factors, and LEP and PON1 as protective factors, affecting the immune cell infiltration level and the levels of proinflammatory factors in glioma. Jiang et al. showed a correlation between TERT alterations and tumor immune microenvironment, and thus suggested that TERT can serve as potential biomarkers for individualized immunotherapy [41]. LEP, shown to play a crucial role in the initiation of the immune system, had also been indicated to be one of the mediators of inflammation responsible [42, 43]. Aharoni et al. showed PON1 to have an important function as an anti-inflammatory, which could reduce sustained pro-inflammatory responses [44]. While there are few studies reported the association between the SSTR3 expression and immune microenvironment in human cancer.

In our study, we developed a risk model with four selected AGs, and the results revealed that the risk score had a prognostic value and was associated with tumor immune microenvironment in glioma. Nevertheless, there were still some limitations of our study that needed to be overcome. First, the bioinformatics results were validated based on the TCGA and CGGA datasets The relationship between the risk signature of four survival-associated AGs and clinical prognostic value of patient with glioma were not subjected to external verification due to the lack of our own adequate available data; external validation should be performed based on our own data in the future. Second, we only performed the RT-qPCR and immunohistochemistry assay to substantiate our bioinformatics results; we should further perform more laboratory experiments to confirm this conclusion.

In conclusion, we established a risk model with four selected AGs, which showed potential function as immune checkpoint inhibitors and hence promise as targets for developing individualized immunotherapy for patient with glioma.

## MATERIALS AND METHODS

### Data collection and acquisition

The mRNA expression files and related clinicopathological data were acquired from the TCGA (n = 628) (https://portal.gdc.cancer.gov/) and CGGA (https://www.cgga.org.cn/) (n = 620)) datasets (Supplementary Table 2). A list of AGs was acquired from the human aging genome resource dataset (http://genomics.senescence.info/genes/) [45], and copy number alterations data were acquired from cBioPortal websites (http://www.cbioportal.org/) [46].

### Selected survival-associated AGs and investigation of their potential cellular biological effects

We identified differentially expressed genes between LGG and GBM samples, and selected the differentially expressed AGs from differentially expressed genes based on TCGA datasets, according to the standards of | log2(Fold change) | > 1 and *P* < 0.05. We selected survival-associated AGs according to the standard of *P* < 0.01 and | hazard ratio | > 1 by univariate Cox analysis using the R package “survival”. Then we sorted patients with glioma into two clusters basing on the four selected AGs expression profiles of samples from TCGA datasets using the R package “ConsensusClusterPlus”. The Euclidean distance was used to compute the similarity distance between patients with glioma, and the k-means method was utilized for clustering based on 50 iterations, which each iteration included 80% of patients. The optimal number of clusters was identified by CDF and consensus matrices. Then we validated the classification results by performing PCA. The molecular mechanisms of selected survival-associated AGs were explored by performing GO pathway analyses, KEGG pathway analyses, and GSEA.

### Establishing a risk model and exploring the prognostic value of risk score

To further investigate the biological functions of the four selected survival-associated AGs, we performed Lasso Cox regression algorithm based on the expression levels of survival-associated AGs, and four selected AGs were identified based on the minimum criteria to establish the risk score (Supplementary Figure 2). Each risk score was defined by the coefficients derived from Lasso algorithm, and the formula was as followed: risk score = [TERT expression * (0.226371)] + [LEP expression * (0.30102)] + [PON1 expression * (-0.34145)] + [SSTR3 expression * (-0.08439)]. We calculated the risk score for glioma sample both in training and validation datasets. We then sorted the glioma samples into two high-risk and low-risk subtypes. A cluster heat map was constructed to display the correlations between candidate genes and risk scores. And ROC curves were constructed to evaluate the prediction efficiency of the risk model. We also constructed nomograms using the R package “rms” and evaluated their performance. The relationships between clinical characteristics and risk score were explored both in training and validation datasets.

### Determination of associations between the level of immune infiltrates, PD-L1 expression, proinflammatory factors, and the prognostic model

We evaluated immune scores of glioma samples using the estimate formula [47]. We calculated the immune and estimate scores according to gene expression profiles and determined their associations with the risk score and the four selected AGs. An estimation of the compositional fraction of 22 types of immune cells of each sample was calculated using CIBERSORT (http://cibersort.stanford.edu/), a tool developed to calculate cellular compositions of tumors according to gene expression data in the TCGA datasets [48]. The relationship between the prognostic model, PD-L1 expression and some proinflammatory factors were investigated based on TCGA datasets, and we normalized the gene expression in the TCGA datasets using formula: log_2_ (N+1).

### Use of RT-qPCR and immunohistochemistry assay to validate bioinformatics results

We collected NBT and glioma tissues specifically 6 NBTs, 10 LGG tissues and 9 GBM tissues from the Second Affiliated Hospital of Nanchang University from May 2017 to June 2020 (Supplementary Table 3). Our study was approved by the Ethics Committee of this hospital. RT-qPCR was conducted using a LightCycler® 480 real-time PCR System based on the manufacturer’s instructions. The expression levels of the four selected AGs were calculated using the 2-ΔΔCt method. Primer sequences for the four AGs were as follows: LEP forward 5’-GCAGTTGCGCAAGTTGTGAT-3’ and reverse 5’-GATGGGCTTCTTGGGCCTTG-3’; TERT forward 5’- CTTGCGGAAGACAGTGGTGA -3’ and reverse 5’-TCCGGGCATAGCTGGAGTA-3’; PON1 forward 5’-CACCCGATGGCAAGTATGTCT-3’ and reverse 5’-GGCATCCAACCCAAAGGTCT-3’; SSTR3 forward 5’-CCCCATGGGCAGGCAAATA-3’ and reverse 5’-CGAGGAGGCATTCTCAGGTT-3’. We performed the immunohistochemistry assay on human tissues by methods described previously [49].

### Statistical analyses

KM curves were used to contrast OS between pairs of subtypes. The Lasso Cox regression formula was used to establish a risk model. Wilcoxon test was used to compare the risk score and four AGs between pairs of subtypes in the different clinical characteristics and PD-L1 expression. Univariate and multivariate Cox regression analyses were used to determine the independent prognostic value of the risk score and the nomogram. The statistical analyses were carried out using R programming language v3.6.3, SPSS Statistics software 26.0, and Prism 8.0.

### Data availability statement

The data for the study were acquired from the TCGA and CGGA datasets and the human aging genome resource.

## Supplementary Material

Supplementary Figures

Supplementary Tables

## References

[1] Jung E, Osswald M, Ratliff M, Dogan H, Xie R, Weil S, Hoffmann DC, Kurz FT, Kessler T, Heiland S, von Deimling A, Sahm F, Wick W, Winkler F. Tumor cell plasticity, heterogeneity, and resistance in crucial microenvironmental niches in glioma. Nat Commun. 2021; 12:1014. 10.1038/s41467-021-21117-3 33579922 PMC7881116

[2] Cloughesy TF, Petrecca K, Walbert T, Butowski N, Salacz M, Perry J, Damek D, Bota D, Bettegowda C, Zhu JJ, Iwamoto F, Placantonakis D, Kim L, et al. Effect of Vocimagene Amiretrorepvec in Combination With Flucytosine vs Standard of Care on Survival Following Tumor Resection in Patients With Recurrent High-Grade Glioma: A Randomized Clinical Trial. JAMA Oncol. 2020; 6:1939–46. 10.1001/jamaoncol.2020.3161 33119048 PMC7596685

[3] Sollmann N, Gutbrod-Fernandez M, Burian E, Riederer I, Meyer B, Hock A, Gempt J, Zimmer C, Kirschke JS. Subtraction Maps Derived from Longitudinal Magnetic Resonance Imaging in Patients with Glioma Facilitate Early Detection of Tumor Progression. Cancers (Basel). 2020; 12:3111. 10.3390/cancers12113111 33114383 PMC7692500

[4] Deland K, Starr BF, Mercer JS, Byemerwa J, Crabtree DM, Williams NT, Luo L, Ma Y, Chen M, Becher OJ, Kirsch DG. Tumor genotype dictates radiosensitization after Atm deletion in primary brainstem glioma models. J Clin Invest. 2021; 131:e142158. 10.1172/JCI142158 32990677 PMC7773366

[5] Phillips RE, Soshnev AA, Allis CD. Epigenomic Reprogramming as a Driver of Malignant Glioma. Cancer Cell. 2020; 38:647–60. 10.1016/j.ccell.2020.08.008 32916125 PMC8248764

[6] Liu Y, Wang Z, Li X, Ma X, Wang S, Kang F, Yang W, Ma W, Wang J. Near-Infrared Fluorescent Peptides with High Tumor Selectivity: Novel Probes for Image-Guided Surgical Resection of Orthotopic Glioma. Mol Pharm. 2019; 16:108–17. 10.1021/acs.molpharmaceut.8b00888 30517013

[7] Wang DD, Deng H, Hervey-Jumper SL, Molinaro AA, Chang EF, Berger MS. Seizure Outcome After Surgical Resection of Insular Glioma. Neurosurgery. 2018; 83:709–18. 10.1093/neuros/nyx486 29126238 PMC6454798

[8] Chiavellini P, Canatelli-Mallat M, Lehmann M, Gallardo MD, Herenu CB, Cordeiro JL, Clement J, Goya RG. Aging and rejuvenation - a modular epigenome model. Aging (Albany NY). 2021; 13:4734–46. 10.18632/aging.202712 33627519 PMC7950254

[9] López-Otín C, Blasco MA, Partridge L, Serrano M, Kroemer G. The hallmarks of aging. Cell. 2013; 153:1194–217. 10.1016/j.cell.2013.05.039 23746838 PMC3836174

[10] Mahmoudi S, Xu L, Brunet A. Turning back time with emerging rejuvenation strategies. Nat Cell Biol. 2019; 21:32–43. 10.1038/s41556-018-0206-0 30602763 PMC7653017

[11] Lee S, Schmitt CA. The dynamic nature of senescence in cancer. Nat Cell Biol. 2019; 21:94–101. 10.1038/s41556-018-0249-2 30602768

[12] Faget DV, Ren Q, Stewart SA. Unmasking senescence: context-dependent effects of SASP in cancer. Nat Rev Cancer. 2019; 19:439–53. 10.1038/s41568-019-0156-2 31235879

[13] Liu XL, Ding J, Meng LH. Oncogene-induced senescence: a double edged sword in cancer. Acta Pharmacol Sin. 2018; 39:1553–58. 10.1038/aps.2017.198 29620049 PMC6289471

[14] Chen Z, Trotman LC, Shaffer D, Lin HK, Dotan ZA, Niki M, Koutcher JA, Scher HI, Ludwig T, Gerald W, Cordon-Cardo C, Pandolfi PP. Crucial role of p53-dependent cellular senescence in suppression of Pten-deficient tumorigenesis. Nature. 2005; 436:725–30. 10.1038/nature03918 16079851 PMC1939938

[15] Braig M, Lee S, Loddenkemper C, Rudolph C, Peters AH, Schlegelberger B, Stein H, Dörken B, Jenuwein T, Schmitt CA. Oncogene-induced senescence as an initial barrier in lymphoma development. Nature. 2005; 436:660–65. 10.1038/nature03841 16079837

[16] Coppé JP, Kauser K, Campisi J, Beauséjour CM. Secretion of vascular endothelial growth factor by primary human fibroblasts at senescence. J Biol Chem. 2006; 281:29568–74. 10.1074/jbc.M603307200 16880208

[17] Liu D, Hornsby PJ. Senescent human fibroblasts increase the early growth of xenograft tumors via matrix metalloproteinase secretion. Cancer Res. 2007; 67:3117–26. 10.1158/0008-5472.CAN-06-3452 17409418

[18] Rao SG, Jackson JG. SASP: Tumor Suppressor or Promoter? Yes!. Trends Cancer. 2016; 2:676–87. 10.1016/j.trecan.2016.10.001 28741506

[19] Ovadya Y, Landsberger T, Leins H, Vadai E, Gal H, Biran A, Yosef R, Sagiv A, Agrawal A, Shapira A, Windheim J, Tsoory M, Schirmbeck R, et al. Impaired immune surveillance accelerates accumulation of senescent cells and aging. Nat Commun. 2018; 9:5435. 10.1038/s41467-018-07825-3 30575733 PMC6303397

[20] Loeser RF, Collins JA, Diekman BO. Ageing and the pathogenesis of osteoarthritis. Nat Rev Rheumatol. 2016; 12:412–20. 10.1038/nrrheum.2016.65 27192932 PMC4938009

[21] Tchkonia T, Zhu Y, van Deursen J, Campisi J, Kirkland JL. Cellular senescence and the senescent secretory phenotype: therapeutic opportunities. J Clin Invest. 2013; 123:966–72. 10.1172/JCI64098 23454759 PMC3582125

[22] Mayne K, White JA, McMurran CE, Rivera FJ, de la Fuente AG. Aging and Neurodegenerative Disease: Is the Adaptive Immune System a Friend or Foe? Front Aging Neurosci. 2020; 12:572090. 10.3389/fnagi.2020.572090 33173502 PMC7538701

[23] Michaud M, Balardy L, Moulis G, Gaudin C, Peyrot C, Vellas B, Cesari M, Nourhashemi F. Proinflammatory cytokines, aging, and age-related diseases. J Am Med Dir Assoc. 2013; 14:877–82. 10.1016/j.jamda.2013.05.009 23792036

[24] Hu MY, Lin YY, Zhang BJ, Lu DL, Lu ZQ, Cai W. Update of inflammasome activation in microglia/macrophage in aging and aging-related disease. CNS Neurosci Ther. 2019; 25:1299–307. 10.1111/cns.13262 31729181 PMC6887669

[25] Kuilman T, Michaloglou C, Vredeveld LC, Douma S, van Doorn R, Desmet CJ, Aarden LA, Mooi WJ, Peeper DS. Oncogene-induced senescence relayed by an interleukin-dependent inflammatory network. Cell. 2008; 133:1019–31. 10.1016/j.cell.2008.03.039 18555778

[26] Geng P, Zhao X, Ou J, Li J, Sa R, Liang H. TERT Genetic Mutations as Prognostic Marker in Glioma. Mol Neurobiol. 2017; 54:3665–69. 10.1007/s12035-016-9930-2 27206431

[27] Yuan Y, Qi C, Maling G, Xiang W, Yanhui L, Ruofei L, Yunhe M, Jiewen L, Qing M. TERT mutation in glioma: Frequency, prognosis and risk. J Clin Neurosci. 2016; 26:57–62. 10.1016/j.jocn.2015.05.066 26765760

[28] Park J, Lee S, Kim K, Park H, Ki CS, Oh YL, Shin JH, Kim JS, Kim SW, Chung JH, Kim TH. TERT Promoter Mutations and the 8th Edition TNM Classification in Predicting the Survival of Thyroid Cancer Patients. Cancers (Basel). 2021; 13:648. 10.3390/cancers13040648 33562809 PMC7915040

[29] Huang FW, Hodis E, Xu MJ, Kryukov GV, Chin L, Garraway LA. Highly recurrent TERT promoter mutations in human melanoma. Science. 2013; 339:957–59. 10.1126/science.1229259 23348506 PMC4423787

[30] Yeh WL, Lu DY, Lee MJ, Fu WM. Leptin induces migration and invasion of glioma cells through MMP-13 production. Glia. 2009; 57:454–64. 10.1002/glia.20773 18814267

[31] Zhang TJ, Xu ZJ, Gu Y, Ma JC, Wen XM, Zhang W, Deng ZQ, Qian J, Lin J, Zhou JD. Identification and validation of obesity-related gene LEP methylation as a prognostic indicator in patients with acute myeloid leukemia. Clin Epigenetics. 2021; 13:16. 10.1186/s13148-021-01013-9 33485366 PMC7824952

[32] Li X, Yu Q. PON1 hypermethylation is associated with progression of renal cell carcinoma. J Cell Mol Med. 2019; 23:6646–57. 10.1111/jcmm.14537 31400051 PMC6787518

[33] Ding GY, Zhu XD, Ji Y, Shi GM, Shen YH, Zhou J, Fan J, Sun HC, Huang C. Serum PON1 as a biomarker for the estimation of microvascular invasion in hepatocellular carcinoma. Ann Transl Med. 2020; 8:204. 10.21037/atm.2020.01.44 32309351 PMC7154400

[34] Hu C, Yi C, Hao Z, Cao S, Li H, Shao X, Zhang J, Qiao T, Fan D. The effect of somatostatin and SSTR3 on proliferation and apoptosis of gastric cancer cells. Cancer Biol Ther. 2004; 3:726–30. 10.4161/cbt.3.8.962 15197339

[35] O’Neil LJ, Kaplan MJ. Neutrophils in Rheumatoid Arthritis: Breaking Immune Tolerance and Fueling Disease. Trends Mol Med. 2019; 25:215–27. 10.1016/j.molmed.2018.12.008 30709614

[36] Mantovani A, Cassatella MA, Costantini C, Jaillon S. Neutrophils in the activation and regulation of innate and adaptive immunity. Nat Rev Immunol. 2011; 11:519–31. 10.1038/nri3024 21785456

[37] Rosales C. Neutrophils at the crossroads of innate and adaptive immunity. J Leukoc Biol. 2020; 108:377–96. 10.1002/JLB.4MIR0220-574RR 32202340

[38] Boe DM, Boule LA, Kovacs EJ. Innate immune responses in the ageing lung. Clin Exp Immunol. 2017; 187:16–25. 10.1111/cei.12881 27711979 PMC5167032

[39] Birch J, Gil J. Senescence and the SASP: many therapeutic avenues. Genes Dev. 2020; 34:1565–76. 10.1101/gad.343129.120 33262144 PMC7706700

[40] He S, Sharpless NE. Senescence in Health and Disease. Cell. 2017; 169:1000–11. 10.1016/j.cell.2017.05.015 28575665 PMC5643029

[41] Jiang T, Jia Q, Fang W, Ren S, Chen X, Su C, Zhang L, Zhou C. Pan-cancer analysis identifies TERT alterations as predictive biomarkers for immune checkpoint inhibitors treatment. Clin Transl Med. 2020; 10:e109. 10.1002/ctm2.109 32564494 PMC7403829

[42] Grases-Pintó B, Torres-Castro P, Marín-Morote L, Abril-Gil M, Castell M, Rodríguez-Lagunas MJ, Pérez-Cano FJ, Franch À. Leptin and EGF Supplementation Enhance the Immune System Maturation in Preterm Suckling Rats. Nutrients. 2019; 11:2380. 10.3390/nu11102380 31590415 PMC6836246

[43] Abella V, Scotece M, Conde J, Pino J, Gonzalez-Gay MA, Gómez-Reino JJ, Mera A, Lago F, Gómez R, Gualillo O. Leptin in the interplay of inflammation, metabolism and immune system disorders. Nat Rev Rheumatol. 2017; 13:100–09. 10.1038/nrrheum.2016.209 28053336

[44] Aharoni S, Aviram M, Fuhrman B. Paraoxonase 1 (PON1) reduces macrophage inflammatory responses. Atherosclerosis. 2013; 228:353–61. 10.1016/j.atherosclerosis.2013.03.005 23582715

[45] Tacutu R, Thornton D, Johnson E, Budovsky A, Barardo D, Craig T, Diana E, Lehmann G, Toren D, Wang J, Fraifeld VE, de Magalhães JP. Human Ageing Genomic Resources: new and updated databases. Nucleic Acids Res. 2018; 46:D1083–90. 10.1093/nar/gkx1042 29121237 PMC5753192

[46] Cerami E, Gao J, Dogrusoz U, Gross BE, Sumer SO, Aksoy BA, Jacobsen A, Byrne CJ, Heuer ML, Larsson E, Antipin Y, Reva B, Goldberg AP, et al. The cBio cancer genomics portal: an open platform for exploring multidimensional cancer genomics data. Cancer Discov. 2012; 2:401–04. 10.1158/2159-8290.CD-12-0095 22588877 PMC3956037

[47] Yoshihara K, Shahmoradgoli M, Martínez E, Vegesna R, Kim H, Torres-Garcia W, Treviño V, Shen H, Laird PW, Levine DA, Carter SL, Getz G, Stemke-Hale K, et al. Inferring tumour purity and stromal and immune cell admixture from expression data. Nat Commun. 2013; 4:2612. 10.1038/ncomms3612 24113773 PMC3826632

[48] Newman AM, Liu CL, Green MR, Gentles AJ, Feng W, Xu Y, Hoang CD, Diehn M, Alizadeh AA. Robust enumeration of cell subsets from tissue expression profiles. Nat Methods. 2015; 12:453–57. 10.1038/nmeth.3337 25822800 PMC4739640

[49] Hu LP, Zhang XX, Jiang SH, Tao LY, Li Q, Zhu LL, Yang MW, Huo YM, Jiang YS, Tian GA, Cao XY, Zhang YL, Yang Q, et al. Targeting Purinergic Receptor P2Y2 Prevents the Growth of Pancreatic Ductal Adenocarcinoma by Inhibiting Cancer Cell Glycolysis. Clin Cancer Res. 2019; 25:1318–30. 10.1158/1078-0432.CCR-18-2297 30420446

