# Supplementary material for: A risk signature of four aging-related genes has clinical prognostic value and is associated with a tumor immune microenvironment in glioma
Source: Aging (Albany NY). 2021 Jun 10;13(12):16198–218. doi: 10.18632/aging.203146 (PMC8266313; doi:10.18632/aging.203146)
Supplement: Supplementary Figures [file aging-13-203146-s001.pdf]

SUPPLEMENTARY FIGURES

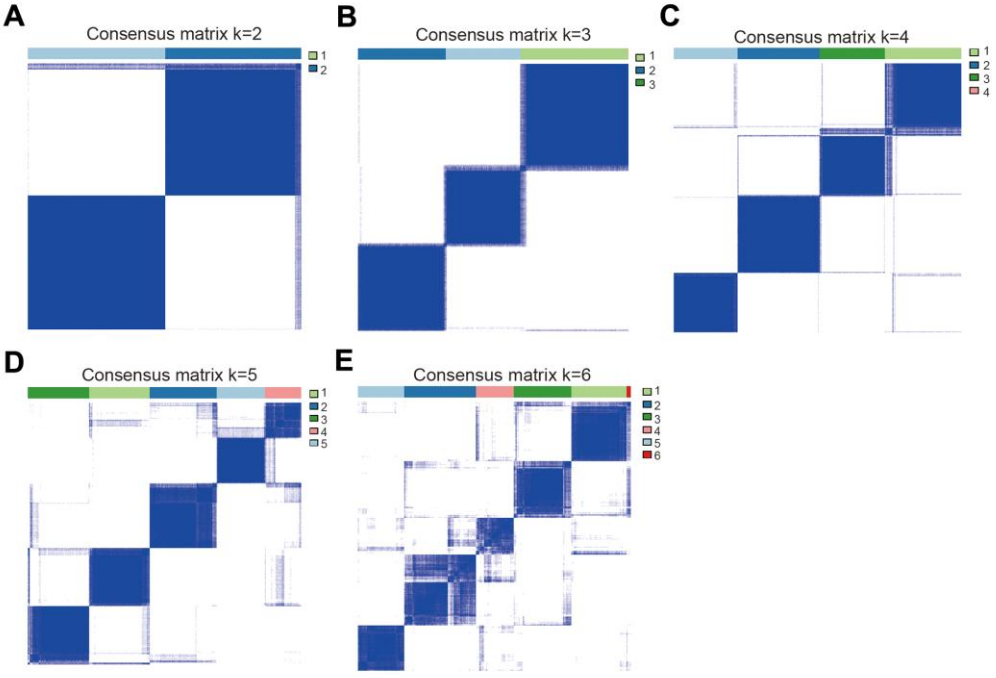

Supplementary Figure 1. The consensus score matrix of the four survival-associated AGs expression in glioma samples based on TCGA datasets. The consensus score matrix of the four survival-associated AGs expression in glioma samples based on TCGA datasets. (**A–E**) at K = 2 (**A**), 3 (**B**), 4 (**C**), 5 (**D**), and 6 (**E**), the correlation between groups, the consensus score matrix for the four selected AGs in the TCGA datasets.

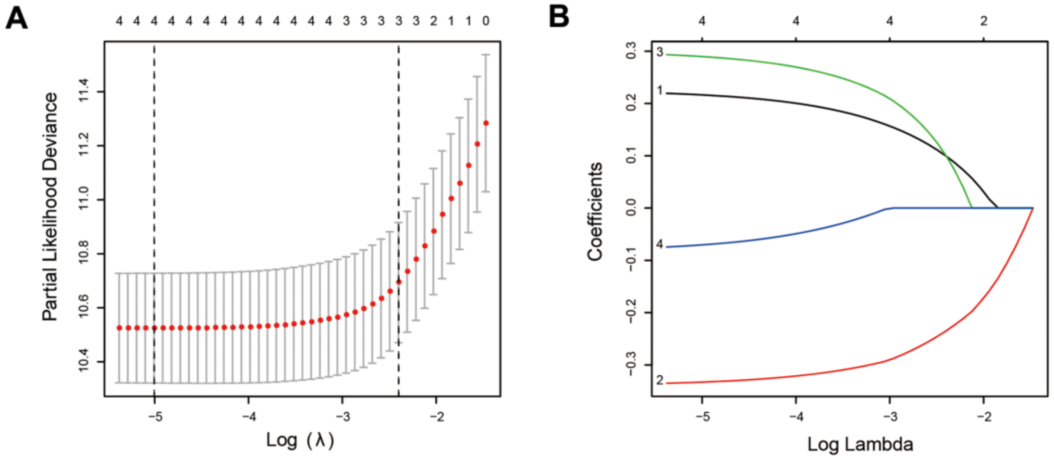

Supplementary Figure 2. Risk model derived from expression patterns of four survival-associated AGs. (**A**) Partial likelihood deviance for tuning the parameter selection in the Lasso regression model in the TCGA datasets. (**B**) Lasso coefficient profiles of the four survival-associated AGs in the TCGA datasets.

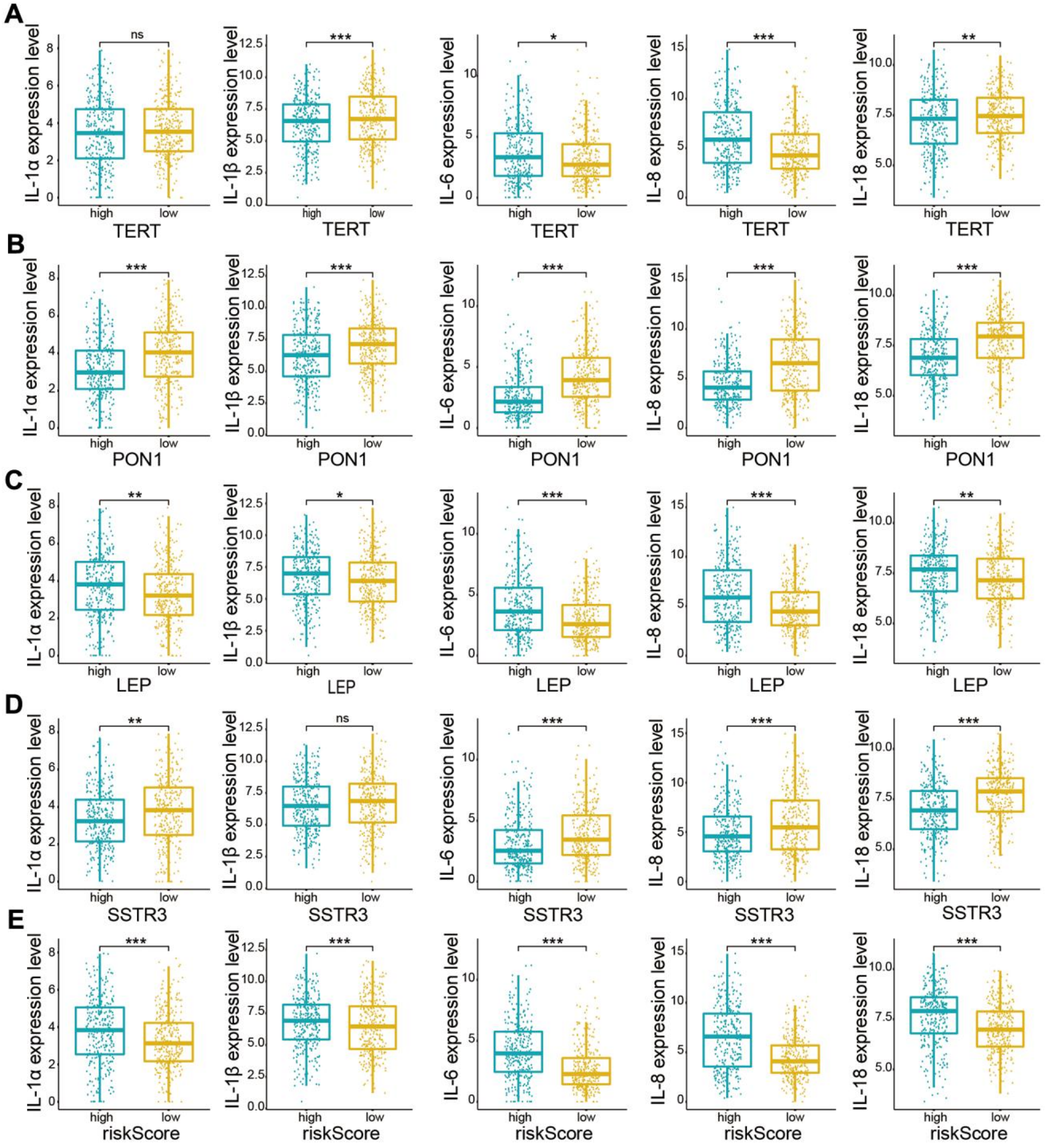

Supplementary Figure 3. The expressing features of interferons and chemokines based on expression levels of the four selected AGs and the risk score. (**A**–**E**) Distribution of interferons and chemokines (IL-1α, IL-1β, IL-6, IL-8, and IL-18) expression levels based on expression levels of the four selected AGs (TERT (**A**), PON1(**B**), LEP (**C**), and SSTR3 (**D**)) of patients with glioma and the risk score (**E**) in the TCGA datasets.
