# Supplementary material for: A risk signature of four aging-related genes has clinical prognostic value and is associated with a tumor immune microenvironment in glioma
Source: Aging (Albany NY). 2021 Jun 10;13(12):16198–218. doi: 10.18632/aging.203146 (PMC8266313; doi:10.18632/aging.203146)
Supplement: Supplementary Tables [file aging-13-203146-s002.pdf]

SUPPLEMENTARY TABLES

Supplementary Table 1. Clinicopathological features of the clusters included in this study.

TCGA dataset			
	**Cluster1**	**Cluster2**	***P*-value**
Total cases	317	311	
Sex			0.742
Male	179	183	
Female	138	128	
Age			<0.001
<50	229	121	
>=50	88	190	
Grade			<0.001
II	182	37	
III	123	120	
IV	12	154	
IDH			<0.001
Mutation	276	108	
Wildtype	39	196	
NA	2	7	
1p19q			<0.001
Codel	110	42	
Non-codel	207	262	
NA	0	6	
Survival state			<0.001
Alive	50	149	
Dead	267	162	

Supplementary Table 2. Clinicopathological features of patients included in this study.

	TCGA dataset		CGGA dataset	
	**Number**	**Percentage**	**Number**	**Percentage**
**Total**	628	100%	620	100%
**Age**	14-89 (47)		11-76 (43)	
<median	308	49.04%	320	51.61%
≥median	320	50.96%	299	48.22%
NA	0	0%	1	0.17%
**Gender**				
Female	266	42.36%	264	42.58%
Male	362	51.91%	356	57.42%
NA	0	0%	0	0%
**Grade**				
WHO II	219	34.87%	173	27.9%
WHO III	243	38.69%	232	37.42%
WHO IV	156	24.84%	215	34.68%
NA	0	0%	0	0%
**IDH**				
Wildtype	235	37.42%	258	41.61%
Mutation	384	61.15%	317	51.13%
NA	9	1.43%	45	7.26%
**1p/19q**				
Non-codel	470	74.84%	428	69.03%
Codel	152	24.20%	128	20.65%
NA	6	0.96%	64	10.32%

Supplementary Table 3. Clinical data of the patients that used in the RT-qPCR and immunohistochemistry assay.

Number	Age-ranges (years old)	Gender	paired/unpaired	type of tissue	Hospitalization date
1	40-50	Male	unpaired	NBT	2019.05.25
2	70-80	Female	unpaired	NBT	2018.09.12
3	60-70	Male	unpaired	NBT	2018.03.12
4	30-40	Male	unpaired	NBT	2020.02.19
5	60-70	Female	unpaired	NBT	2017.07.08
6	70-80	Male	unpaired	NBT	2018.04.29
7	60-70	Female	unpaired	LGG	2019.09.17
8	50-60	Male	unpaired	LGG	2018.07.12
9	30-40	Female	unpaired	LGG	2019.01.27
10	70-80	Female	unpaired	LGG	2018.10.14
11	40-50	Male	unpaired	LGG	2018.03.28
12	20-30	Male	unpaired	LGG	2020.02.02
13	50-60	Male	unpaired	LGG	2018.01.23
14	40-50	Female	unpaired	LGG	2017.11.29
15	60-70	Female	unpaired	LGG	2017.09.27
16	50-60	Male	unpaired	LGG	2019.05.18
17	30-40	Male	unpaired	GBM	2017.12.21
18	40-50	Female	unpaired	GBM	2019.02.17
19	50-60	Male	unpaired	GBM	2019.01.07
20	50-60	Female	unpaired	GBM	2018.11.28
21	60-70	Male	unpaired	GBM	2018.04.19
22	40-50	Female	unpaired	GBM	2017.10.14
23	70-80	Male	unpaired	GBM	2018.07.27
24	60-70	Male	unpaired	GBM	2019.02.13
25	60-70	Female	unpaired	GBM	2018.12.25
